# Supplementary material for: Pain perception genes, asthma, and oral health: A reverse genetics study
Source: PLoS One. 2022 Nov 17;17(11):e0277036. doi: 10.1371/journal.pone.0277036 (PMC9671307; doi:10.1371/journal.pone.0277036)
Supplement: Attachment — Submitted filename: PLOS ONE - Comments to the author.docx [file pone.0277036.s002.docx]

1. Was only one type of anesthetic compared? Example: lidocaine with vasoconstrictor. And in similar clinical situations? Example: in third molar extractions or class I restorations or endodontic treatment, etc. It is necessary to describe what types of dental treatment were used as a parameter in anesthesia, and what anesthetics were used.

2. In table 01, include what the numbers inside the parentheses mean (maximum and minimum?). If so, it is important to distinguish adults from children, since the parameter used to evaluate the groups was the amount of anesthetic during the dental procedure; in children, fear and anxiety greatly influence pain, especially during procedures involving needles, as is the case with anesthesia.

3. Was it the same person who anesthetized all participants? Has any calibration been done between dentists? What kind of treatment was done on everyone to compare anesthesia?

4. The presence of some inflamed tissue can influence the amount of anesthetic tubes to be used in each procedure, was this taken into account?

5. How were these phenotypes evaluated? More detail is needed.

6. It is necessary to mention which studies were used as a reference in the choice of SNPs.

7. It is necessary to include the background of the selected SNPs in the introduction.

8. Add in table 02 information such as “increase or decrease pain sensitivity” other than just “Intensity or pain sensitivity”. So we would know what exactly the polymorphism effect on pain perception.

9. Was an epistasis analysis performed? Some studies demonstrate that the GCH1 gene can modulate the effect of COMT on the pain response. Patients who have SNP in both genes usually have a protective effect (analgesia) by GCH1, even with the COMT SNP still present.

10. I suggest increasing the quality of the images, it is difficult to understand what is written.

11. Results need to be organized more clearly, it's confusing.

12. I suggest further specifying the purpose of the work. Through the methodology of the work, the objective was to investigate these clinical phenotypes and some genetic markers in the response/efficacy of anesthetic or analgesia and not necessarily pain. It is necessary to clarify the justification and background of these objectives during the introduction, as it is not clear exactly what this work is about, only during the reading of the methodology. There are numerous ways to work with or assess pain: acute, spontaneous, chronic, stimulated/induced. It is necessary to specify and better describe the type of pain being investigated in this study, since one of the objectives is precisely to make this correlation between pain-related SNPs and other clinical phenotypes.

13. Importantly, the SNPs investigated here are not exclusively related to nociceptive pathways, but also to other non-painful conditions. It is necessary to describe each one of them better in this sense, depending on the amount of clinical phenotypes that were included in the analysis and are not directly related to nociceptive processes. Furthermore, it is necessary to include this observation in the discussion of the work.

14. The title is a few confusing: “Pain Perception Genes, Asthma, and Oral Health: A Reverse Genetics Study”. What does it mean “reverse genetics study” ?

15. The only relationship with pain in this work was the way the sample was divided into two groups – and this division, and the genes that are really related to painful processes, still need to be more detailed. However, the immense amount of clinical phenotypes evaluated are not directly related to pain, it is necessary to establish a link between these phenomena and describe them very well in the study.

16. It should also be discussed that there was not found any difference between the both groups after Bonferroni correction. Perhaps the method of using the amount of anesthetic as a parameter for dividing the groups was not satisfactory for evaluating these clinical phenotypes (pain-related? What exactly?) with genetic markers of pain. There are many factors that influence the amount of anesthetic used. This was a decisive criterion in the selection of the two groups, however, the way in which it was made was neither mentioned nor described. If there was calibration between the dentists who performed the procedure (both anesthetic and dental – surgical/restorative/endodontic/periodontal – which should also be described), nor if there was any nervous branch of predilection and region that was anesthetized – maxilla or mandible, if there was previous pain or local inflammation/infection. These are all factors that influence the anesthesia procedure, and that influenced the choice/division of the study sample.

17. The final considerations state that “Nevertheless, our study provides a pioneering approach to the study of pain perception”. What tool was used to evaluate pain perception? Visual analogue scale (VAS)? Sound, eye, and motor (SEM) indexes? Quantitative Sensory Testing?

18. Which previous study was used to determine the cut-off point for the amount of anesthetic? So it could be used as parameter to dived the sample into two groups.
