# Supplementary material for: Pain perception genes, asthma, and oral health: A reverse genetics study
Source: PLoS One. 2022 Nov 17;17(11):e0277036. doi: 10.1371/journal.pone.0277036 (PMC9671307; doi:10.1371/journal.pone.0277036)
Supplement: Attachment — Submitted filename: Response to Reviewers.docx [file pone.0277036.s003.docx]

**Journal Requirements:**

**When submitting your revision, we need you to address these additional requirements.**

**1. Please ensure that your manuscript meets PLOS ONE's style requirements, including those for file naming. The PLOS ONE style templates can be found at**

**https://journals.plos.org/plosone/s/file?id=wjVg/PLOSOne_formatting_sample_main_body.pdf and**

[**https://journals.plos.org/plosone/s/file?id=ba62/PLOSOne_formatting_sample_title_authors_affiliations.pdf**](https://nam12.safelinks.protection.outlook.com/?url=https%3A%2F%2Fjournals.plos.org%2Fplosone%2Fs%2Ffile%3Fid%3Dba62%2FPLOSOne_formatting_sample_title_authors_affiliations.pdf&data=05%7C01%7Carv11%40pitt.edu%7C7645f2399862427ae89c08da5dace205%7C9ef9f489e0a04eeb87cc3a526112fd0d%7C1%7C0%7C637925295632411057%7CUnknown%7CTWFpbGZsb3d8eyJWIjoiMC4wLjAwMDAiLCJQIjoiV2luMzIiLCJBTiI6Ik1haWwiLCJXVCI6Mn0%3D%7C3000%7C%7C%7C&sdata=PfJ1FLsVxJf2FcaRr7pVrZb4Voq6beu9ptkUf%2FZL6VM%3D&reserved=0)

All information has been revised as requested.

**2. Thank you for stating the following financial disclosure:**

**“The funders had no role in study design, data collection and analysis, decision to publish, or preparation of the manuscript.”**

**At this time, please address the following queries:**

**a)        Please clarify the sources of funding (financial or material support) for your study. List the grants or organizations that supported your study, including funding received from your institution.**

**b)        State what role the funders took in the study. If the funders had no role in your study, please state: “The funders had no role in study design, data collection and analysis, decision to publish, or preparation of the manuscript.”**

**c)        If any authors received a salary from any of your funders, please state which authors and which funders.**

**d)        If you did not receive any funding for this study, please state: “The authors received no specific funding for this work.”**

**Please include your amended statements within your cover letter; we will change the online submission form on your behalf.**

The additional information in the acknowledgment section was excluded. It is marked in yellow and crossed out with a line.

**3. Thank you for stating the following in the Acknowledgments Section of your manuscript:**

**"We would like to acknowledge the Dental Registry and DNA Repository project, which is supported by the University of Pittsburgh School of Dental Medicine. Also, we thank the Graduate Program in Oncology and Medical Sciences at the Federal University of Pará, Brazil, and the National Center for Advancing Translational Sciences of the National Institutes of Health, for the support to Lisboa, RO, and Bezamat, M, respectively.”**

**We note that you have provided additional information within the Acknowledgements Section that is not currently declared in your Funding Statement. Please note that funding information should not appear in the Acknowledgments section or other areas of your manuscript. We will only publish funding information present in the Funding Statement section of the online submission form.**

**Please remove any funding-related text from the manuscript and let us know how you would like to update your Funding Statement. Currently, your Funding Statement reads as follows:**

**“The funders had no role in study design, data collection and analysis, decision to publish, or preparation of the manuscript.”**

**Please include your amended statements within your cover letter; we will change the online submission form on your behalf.**

The additional information in the acknowledgment section was excluded. It is marked in yellow and crossed out with a line. We added it in the cover letter as asked.

**4. In your Data Availability statement, you have not specified where the minimal data set underlying the results described in your manuscript can be found. PLOS defines a study's minimal data set as the underlying data used to reach the conclusions drawn in the manuscript and any additional data required to replicate the reported study findings in their entirety. All PLOS journals require that the minimal data set be made fully available. For more information about our data policy, please see http://journals.plos.org/plosone/s/data-availability.**

**Upon re-submitting your revised manuscript, please upload your study’s minimal underlying data set as either Supporting Information files or to a stable, public repository and include the relevant URLs, DOIs, or accession numbers within your revised cover letter. For a list of acceptable repositories, please see http://journals.plos.org/plosone/s/data-availability#loc-recommended-repositories. Any potentially identifying patient information must be fully anonymized.**

**Important: If there are ethical or legal restrictions to sharing your data publicly, please explain these restrictions in detail. Please see our guidelines for more information on what we consider unacceptable restrictions to publicly sharing data: http://journals.plos.org/plosone/s/data-availability#loc-unacceptable-data-access-restrictions. Note that it is not acceptable for the authors to be the sole named individuals responsible for ensuring data access.**

**We will update your Data Availability statement to reflect the information you provide in your cover letter.**

We added a supporting information file with our raw data.

**5. We note that you have stated that you will provide repository information for your data at acceptance. Should your manuscript be accepted for publication, we will hold it until you provide the relevant accession numbers or DOIs necessary to access your data. If you wish to make changes to your Data Availability statement, please describe these changes in your cover letter and we will update your Data Availability statement to reflect the information you provide.**

We changed the cover letter to reflect that we are uploading all raw data as an appendix.

**Reviewers' comments:**

**Reviewer #1:**

**1. Was only one type of anesthetic compared? Example: lidocaine with vasoconstrictor. And in similar clinical situations? Example: in third molar extractions or class I restorations or endodontic treatment, etc. It is necessary to describe what types of dental treatment were used as a parameter in anesthesia, and what anesthetics were used.**

The information of the local anesthetics used were added to the manuscript in the methods section (line 134) and also the duration of anesthesia according to the article reference.

**2. In table 01, include what the numbers inside the parentheses mean (maximum and minimum?). If so, it is important to distinguish adults from children, since the parameter used to evaluate the groups was the amount of anesthetic during the dental procedure; in children, fear and anxiety greatly influence pain, especially during procedures involving needles, as is the case with anesthesia.**

The number inside the parentheses mean the range of age in each group. As you can see in the table 1 the groups were matched for age, so there are no significant variations between groups. Both children and adults may display some fear and anxiety. 90% of pediatric dentistry is done under local anesthesia. Comparison groups were originally matched by age to mitigate the influence of age.

**3. Was it the same person who anesthetized all participants? Has any calibration been done between dentists? What kind of treatment was done on everyone to compare anesthesia?**

Data for the study comes from our registry, that currently spans over 17 years. It was not the same operator that anesthetized all participants. All dentists in training perform anesthesia under the same protocol. Treatments done were typical operative dentistry procedures. We added these clarifications in the Methods section (line 137) to address this concern.

**4. The presence of some inflamed tissue can influence the amount of anesthetic tubes to be used in each procedure, was this taken into account?**

To mitigate this aspect, comparison groups were matched by dental procedure. But beyond that, it was not possible to fully account for levels of inflammation at the day treatments were performed. We added a clarification in the method section (line 139) to acknowledge this detail.

**5. How were these phenotypes evaluated? More detail is needed.**

Phenotypes were defined based on coding used in the registry, which reflects descriptions at the dental records. We added this information in the methods section (line 159) to address this concern.

**6. It is necessary to mention which studies were used as a reference in the choice of SNPs.**

The studies used as references for each SNP were originally mentioned in table 2.

**7. It is necessary to include the background of the selected SNPs in the introduction.**

The background of the SNPs was inserted in the introduction.

**8. Add in table 02 information such as “increase or decrease pain sensitivity” other than just “Intensity or pain sensitivity”. So we would know what exactly the polymorphism effect on pain perception.**

The SNPs in the chosen genes act mainly in the general context of the pain perception process and unfortunately in the literature there are no specific concrete associations of these SNPs regarding their participation in the increase or decrease of pain, many of them seem to act in both situations depending on some factors, therefore, to avoid future mistakes when conditioning them with a specific association, it is preferable to use the term “Intensity or pain sensitivity” as used in the reference articles. Among the suggested explanations for the discrepancy found in the literature is that this may indicate that these polymorphisms affect pain sensations of some, but not all, stimuli. This information will be added to the manuscript to explain why we used the term.

**9. Was an epistasis analysis performed? Some studies demonstrate that the GCH1 gene can modulate the effect of COMT on the pain response. Patients who have SNP in both genes usually have a protective effect (analgesia) by GCH1, even with the COMT SNP still present.**

No. For this specific paper epistasis analysis was not performed because the study's main objective was to search associations using the PheWAS methodology and not the influence that could exist between the studied markers.

**10. I suggest increasing the quality of the images, it is difficult to understand what is written.**

All images were submitted to the PACE tool and the quality of the images was enhanced.

**11. Results need to be organized more clearly, it's confusing.**

The organization was done according to the specific results of each PheWAS analysis performed and following the presentation standard for this methodology.

**12. I suggest further specifying the purpose of the work. Through the methodology of the work, the objective was to investigate these clinical phenotypes and some genetic markers in the response/efficacy of anesthetic or analgesia and not necessarily pain. It is necessary to clarify the justification and background of these objectives during the introduction, as it is not clear exactly what this work is about, only during the reading of the methodology. There are numerous ways to work with or assess pain: acute, spontaneous, chronic, stimulated/induced. It is necessary to specify and better describe the type of pain being investigated in this study, since one of the objectives is precisely to make this correlation between pain-related SNPs and other clinical phenotypes.**

The information in the main sections of the manuscript has been improved for a better understanding of the purpose of the study and how it was done.

**13. Importantly, the SNPs investigated here are not exclusively related to nociceptive pathways, but also to other non-painful conditions. It is necessary to describe each one of them better in this sense, depending on the amount of clinical phenotypes that were included in the analysis and are not directly related to nociceptive processes. Furthermore, it is necessary to include this observation in the discussion of the work.**

Most of phenotypes included in the analysis have a pain sensitivity component. SNPs were chosen because they have some association with the pain perception processor regardless of whether it is nociceptive pain or not. And since the perception of pain is something extremely complex with the involvement of several genes, it is expected that these SNPs participate in other processes and are also associated with other non-painful conditions.

**14. The title is a few confusing: “Pain Perception Genes, Asthma, and Oral Health: A Reverse Genetics Study”. What does it mean “reverse genetics study” ?**

In reverse genetics, genetic marker data are used to drive, or form the basis of, new phenotype definitions, that is, the analysis starts from genotype to phenotype. We addeda clarification in the Introduction section (line 83)/

**15. The only relationship with pain in this work was the way the sample was divided into two groups – and this division, and the genes that are really related to painful processes, still need to be more detailed. However, the immense amount of clinical phenotypes evaluated are not directly related to pain, it is necessary to establish a link between these phenomena and describe them very well in the study.**

Most phenotypes included actually have potentially a pain sensitivity component associated with them. We added this clarification in the methods section (line 162).

**16. It should also be discussed that there was not found any difference between the both groups after Bonferroni correction. Perhaps the method of using the amount of anesthetic as a parameter for dividing the groups was not satisfactory for evaluating these clinical phenotypes (pain-related? What exactly?) with genetic markers of pain. There are many factors that influence the amount of anesthetic used. This was a decisive criterion in the selection of the two groups, however, the way in which it was made was neither mentioned nor described. If there was calibration between the dentists who performed the procedure (both anesthetic and dental – surgical/restorative/endodontic/periodontal – which should also be described), nor if there was any nervous branch of predilection and region that was anesthetized – maxilla or mandible, if there was previous pain or local inflammation/infection. These are all factors that influence the anesthesia procedure, and that influenced the choice/division of the study sample.**

Bonferroni is quite a strict method of multiple comparion correction. One result survived this correction. Results that do not survive a Bonferroni correction were also presented and have been added to the Discussion section, since they may represent true biological relationships and deserve further study. The reviewer's other considerations were supplied in the previous comments.

**17. The final considerations state that “Nevertheless, our study provides a pioneering approach to the study of pain perception”. What tool was used to evaluate pain perception? Visual analogue scale (VAS)? Sound, eye, and motor (SEM) indexes? Quantitative Sensory Testing?**

The final consideration was re-elaborated to suit the proposed objectives and results of the study.

**18. Which previous study was used to determine the cut-off point for the amount of anesthetic? So it could be used as parameter to dived the sample into two groups.**

It is common clinical practice to give up to one tubete of anesthetic for dental treatments. Hence, the cutoff used.

**Reviewer #2: The authors performed PheWAS-like analysis to examine the associations of 58 phenotypes with 7 pain related SNPs. A total of 1289 subjects were selected and grouped based on the dose of anesthesia received during the dental treatment procedure. They found an association between the rs1799971 in OPRM1 and asthma, which was in agreement with a recent report. In addition, the study identified three associations of oral phenotypes with variants of pain-related genes. Overall, the study is carefully designed and performed.**

**Major critiques:**

**1. The authors applied novel criteria to recruit subjects - individuals who feel more pain (received more anesthesia) or less pain (who received less anesthesia). However, using “anesthesia utilization” as a surrogate indicator of pain severity raised concerns. It is unknown if other factors were taken into consideration, e.g. medication history, especially opioids and other analgesic drugs, dental procedure types (location, duration), as well as pain assessment (score, type). Whether these variants were adjusted in the statistical analysis was not mentioned in the manuscript.**

Groups were matched by dental procedures, but chronic use of opioids or other analgesic oral drugs, was not considered. No pain assessments were performed.

**2. The sample size of "black – less anesthesia" group is small. The authors should address how they controlled the power of analysis to ensure the association detected between rs4818 of COMT and diseases of the tongue was not false-positive.**

Test power was controlled using the simulation study by Verma et al. (2018) and according to this study for the group that required less anesthetic, there was sufficient power if the allele frequency was 20% and the penetrance was 20% or higher.

**3. The authors should address the rationale of selecting these 7 SNPs, in terms of their relationships with orofacial pain perception, specifically.**

SNPs were selected for their involvement with the perception of the general participation of genes in fundamental processes. Those SNPs that are related to orofacial pain were described in the introduction for better understanding. Per reviewer 1, rationale was added to the introduction section.

**Minor issues:**

**Line 233, Appendix Table 1 is not included in the manuscript.**

The appendix table1 was inserted in the main text as table 5.

**Line 143, reference is missing.**

The missing reference was added.
