# Supplementary material for: Pain perception genes, asthma, and oral health: A reverse genetics study
Source: PLoS One. 2022 Nov 17;17(11):e0277036. doi: 10.1371/journal.pone.0277036 (PMC9671307; doi:10.1371/journal.pone.0277036)
Supplement: Attachment — Submitted filename: Response to Reviews.docx [file pone.0277036.s004.docx]

Response to Reviews

Reviewer #1: The authors answered most of the questions raised and significantly improved the quality of the manuscript. However, there are still major concerns that need to be addressed.

1. Lidocaine and articaine have very different particularities. For instance, The duration of effect in a maxillary infiltration of 2% lidocaine with 1:100,000 epinephrine ranges from 60 to 90 minutes in pulp tissue and 170 to 300 minutes in soft tissue, while the duration of 4% articaine with 1:100,000 epinephrine encompasses 60 to 75 minutes in the pulp and 170 to 360 minutes in the soft tissues. Palate anesthesia is unpleasant for many patients because it is a very sensitive site, so when the anesthetic used is articaine, it is not necessary to perform infiltration in the palatal location for extraction procedures, as it is very effective only with injection in the region maxillary vestibular. Articaine presents tissue diffusion when compared to the lidocaine in areas other than the place where the anesthetic was deposited, avoiding excessive punctures and patient discomfort. Therefore, depending on the dental procedure, the type of anesthetic could influence the number of tubetes to be used. This should have been considered in the methodological strategy of the study.

RESPONSE: We added discussion to acknowledge this limitation.

2. Regarding including children in the sample, I still have concerns about the parameter of “quantity of anesthetic used”. Since we use weight calculate the maximum amount of anesthetic. In this way, children necessarily must use less tubetes then adults. Since the parameter of “pain perception” is the amount of anesthetic used, these variables involved in metabolization, duration effect, toxicity and others, should be addressed accordingly.

RESPONSE: We added discussion and acknowledged this limitation.

3. The final consideration state that “Through this powerful strategy, it was possible to identify new associations between SNPs in genes related to pain and oral phenotypes in the groups of individuals who feel more pain (those who received more anesthesia) or less pain (who received less anesthesia)”. Actually, it is not possible to establish this type of equivalence. Since the amount of anesthetic used can be influenced by several factors, such as anatomical variations, metabolism rate, type of anesthetic used, local tissue pH (which can be modified by the presence of inflammation), technique used, among others, and not just pain.

RESPONSE: We rewrote the statement to address this concern.

Reviewer #2: The authors have carefully addressed the questions from the reviewers. The revised manuscript is in better shape. However, there are couple places need additional inputs.

First of all, based on the analysis scale (58 phenotypes, 7 SNPs, 1289 subjects), it can not be called as a real PheWAS study. The authors should be careful when using the term in the manuscript.

RESPONSE: The definition of PheWAS is a study design in which the association between single nucleotide polymorphisms or other types of DNA variants is tested across a large number of different phenotypes. Based on the definition, our study is a PheWAS study.

Second, the authors should revise the Introduction to be more concise and focused. This is not a review paper.

RESPONSE: We shortened the introduction as suggested.
