# Supplementary material for: Pain perception genes, asthma, and oral health: A reverse genetics study
Source: PLoS One. 2022 Nov 17;17(11):e0277036. doi: 10.1371/journal.pone.0277036 (PMC9671307; doi:10.1371/journal.pone.0277036)
Supplement: Attachment — Submitted filename: Response to Reviews2.docx [file pone.0277036.s005.docx]

Response to Reviewers:

Reviewer #1: The authors have carefully addressed the questions from the reviewers, and significantly improved the quality of the manuscript. However, there are still minor issues that need to be addressed. All the new data included are lacking references. It is necessary to cite the sources from which the information was obtained.

RESPONSE: We added the references as requested.
